# High Lithium Bioaccumulation and Depuration Rates in Marine Mussels (*Mytilus edulis*): An Isotopic Perspective

**DOI:** 10.1021/acsomega.6c05538

**Published:** 2026-10-22

**Authors:** Dongyu Chen, Théo Tacail, Yuki Mark, François Oberhänsli, Maryline Montanes, Hyeryeong Jeong, Emilia Vassileva, Anna Maria Orani, Laurine Payant, Arbia Jouini, Fanny Thibon, Philippe Telouk, Marc Métian, Nathalie Vigier

**Affiliations:** † Laboratoire d’Océanographie de Villefranche, CNRS, 129692 Sorbonne Université, Villefranche-sur-Mer 06230, France; ‡ 551793 IAEA Radioecology Laboratory, Department of Nuclear Sciences and Applications, Monaco 98000, Principality of Monaco; § The Isotoparium, Division of Geological and Planetary Sciences, California Institute of Technology, Pasadena, California 91125, United States; ∥ Laboratoire de Géologie de Lyon, Ecole Normale Supérieure de Lyon, CNRS UMR 5276, 26911 Université de Lyon, 46 Allée d’Italie, Lyon 69007, France

## Abstract

The growing demand for lithium (Li), driven by its use in rechargeable batteries and energy storage technologies, raises concerns about its environmental impact, especially in coastal ecosystems, which serve as receptacles for many pollutants. This study quantifies the rates of Li uptake and depuration in marine mussels *(Mytilus edulis)*, using both natural variations in Li isotopes and a parameterized box model. Mussels were exposed to Li-enriched seawater (1.5 mg/L) for up to 5 days, followed by a depuration phase in natural seawater (0.18 mg/L). Results reveal rapid uptake, with Li concentrations in soft tissues increasing from 0.85 ± 0.08 μg/g (control) to 2.5 ± 0.6 μg/g (exposed), with 70% depuration occurring within 6 h. Exposed mussels exhibit larger isotopic fractionations compared to controls, highlighting the strong sensitivity of Li isotopes to contamination. This pattern also suggests enhanced Li^+^/Na^+^ transporter activity under elevated environmental Li levels. Comparisons with freshwater mussels (*Dreissena polymorpha*) reveal up to 3-fold higher Li bioaccumulation in low-salinity environments, likely due to reduced Na^+^ competition or kidney-mediated salt regulation. Kinetic isotopic modeling confirms rapid equilibrium of Li fluxes, with large uptake and excretion rates (1630 ± 997 ng/h) that balance within 4.2 ± 3.1 h. This study also demonstrates the influence of mussel dry weight on Li bioconcentration factors. These findings refine the use of mussels as sentinels for monitoring coastal Li contamination, while underscoring the need for further work to elucidate their potential to integrate environmental variations over the long-term.

## Graphical abstract

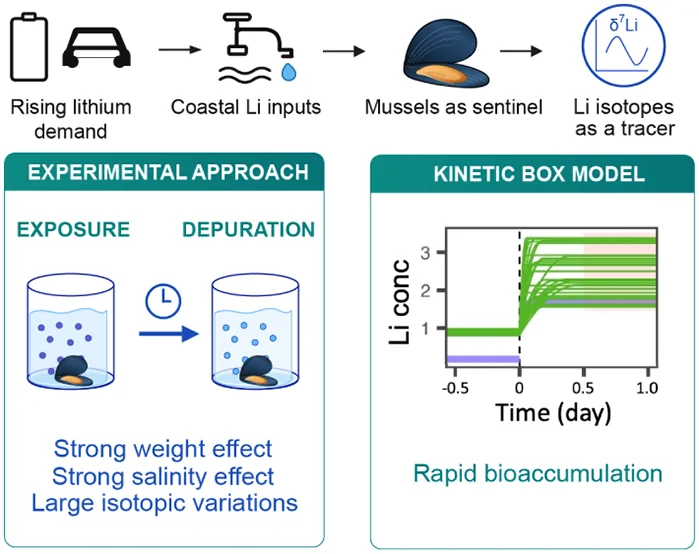

## Introduction

Lithium (Li), an alkali metal with unique physicochemical properties, has become indispensable to modern society. Its very negative electrochemical potential and low atomic weight make it the material of choice for rechargeable Li-ion batteries (LIBs), which now power portable electronics, electric vehicles (EVs), and grid-scale energy storage systems.^1,2^ As global EV production surges amid the energy transition, Li demand has increased rapidly: worldwide consumption reached 134,000 tonnes in 2022,^3^ exceeding the natural riverine input to the ocean (70,000 tonnes) estimated in 1998.^4^ Yet, only 5% of LIBs are recycled,^5^ and less than 10% of global Li is recovered,^2^ leading to risks of anthropogenic Li releases into soils and waterways, including rivers and coastal areas.^6^ Recent studies have already detected elevated Li concentrations in freshwater systems, such as 3,500–7,600 μg/L in Chilean rivers,^7^ primarily sourced from high-altitude Li-rich salt flats. Beyond its industrial dominance, Li plays a critical role in medicine, where Li carbonate remains a cornerstone treatment for bipolar disorder^8,9^ and shows promise in combating neurodegenerative diseases like Alzheimer’s and Parkinson’s.^10,11^ However, despite its widespread industrial and medical use, the environmental fate and biological effects of Li, particularly in coastal ecosystems impacted by riverine inputs, or in basins affected by mining, remain poorly constrained .^12–18^

Mussels and other bivalves are long-established sentinel species for monitoring a wide range of contaminants (e.g., heavy metals, persistent organic pollutants) due to their bioaccumulation capacity, especially in littoral zones.^19^ Nevertheless, Li is rarely included in environmental monitoring programs, and few studies have investigated its ecological effects. Recent research reveals that Li exposure induces metabolic depression, oxidative stress, and neurotoxicity in*Mytilus galloprovincialis* at concentrations as low as 0.75 mg/L.^20^ Sublethal exposure (0.1–10 mg/L) triggers dose-dependent bioaccumulation and histopathological damage, including digestive gland degeneration.^21^ Bioconcentration factors (BCFs)—a key metric for assessing pollutant uptake—vary widely across studies. For instance, BCFs in *Mytilus* range from 1.5 to 6.2, depending on exposure conditions,^22,23^ suggesting that genetic, metabolic, or environmental factors may influence Li accumulation kinetics, as shown for other trace elements.^24–30^ In addition, Na regulation in freshwater and seawater mussels differs, which may lead to different Li BCFs, since Li^+^ is an alkali ion analogous to Na^+^ in biological systems,^34^ but this effect has been little explored so far.

Li isotope analysis (reported as δ^7^Li, in ‰) offers a powerful tool to trace Li sources^31–33^ and elucidate biological processes.^34,35,75^ While δ^7^Li has been extensively applied in geochemistry^36–43^ and paleoclimatology,^44–49^ its potential in biology and ecotoxicology remains underexplored.^22,50–53^

This study aims to bridge these gaps by (a) characterizing Li uptake and depuration kinetics in mussel soft tissues under environmentally relevant conditions, (b) investigating isotopic fractionation patterns to decipher underlying biological mechanisms, and (c) developing a comprehensive model of Li fluxes and isotopic behavior, with implications for addressing the rising threat of anthropogenic Li to coastal ecosystems.

## Materials and Methods

### Sample Preparation

Specimens of the marine bivalve*Mytilus edulis*were collected in June 2019 from La Rochelle, Bay of Biscay, France. After their transfer, the mussels were acclimated for 1 month in 300 L tanks with 1 μm filtered seawater pumped from a depth of 60 m in the Mediterranean Sea (Monaco Bay) (open circuit, water renewal: 300 L/h, temperature: 20.5 ± 0.5 °C, pH_T_: 8.03 ± 0.05, and salinity: 39 ± 1). Continuous aeration ensured water oxygenation, and a 12:12 h light–dark cycle was maintained. All experiments were conducted at the Marine Environmental Laboratory of the International Atomic Energy Agency (IAEA) in Monaco.

### Experimental Conditions

#### Li Exposure and Depuration Experiments

After acclimation, 288 individual mussels were randomly selected and distributed into eight 5 L tanks. Four tanks served as the control group, each filled with 2 L of filtered (<1 μm) seawater, with a natural lithium (Li) concentration of 0.18 mg/L, representative of open ocean.^54^ The remaining four tanks formed the experimental group, with an elevated Li concentration of 1.5 mg/L.

We decided to use this relatively high exposure concentration based on data available for waters located in Li-rich estuaries, such as those in Chile (e.g., the Lluta and Camarones estuaries) and based on previous experimental investigations for the same species (see data compilation in ref 22).

A concentrated Li reference solution (1000 mg/L; ICP standard) was evaporated to dryness in precleaned containers, and the residue was subsequently redissolved in filtered seawater to achieve the target Li concentration. During the 5-day exposure period, three individuals per tank were pooled to form a single composite sample at each time point. An additional sample was collected at the 12 h mark (0.5 days) on the first day to assess short-term effects. To minimize potential contamination from residual high-concentration Li on the mussels’ soft tissues, the sampled mussels were rinsed with filtered natural seawater for 30 min. Once the shells opened naturally, the soft tissues were collected and freeze-dried.

On day 5, the remaining mussels from the exposure stage were transferred back to natural (unenriched) seawater to evaluate Li depuration over time. Samples were collected using the same protocol at 5.25, 6, 7, 8, 10, and 15 days.

Natural seawater, filtered to <1 μm, was used for both the depuration phase and the rinsing tests. It has a lithium (Li) concentration representative of the open ocean (0.18 mg/L). The homogeneity of dissolved Li concentrations in the ocean has been widely documented in numerous studies (e.g., 75), including in the Mediterranean Sea, and aligns with its long oceanic residence time (>1 Myr), which far exceeds the mixing time of oceanic water masses (<2 kyr).

#### Rinsing Tests

After collection, mussel tissues were rinsed with control seawater to remove any residual water potentially trapped in micropores or adsorbed on the surface. This step is essential and is commonly performed for trace metals to prevent contamination or interference from the enriched seawater used during the exposure experiment.

To validate the rinsing methodology for Li and ensure that no biologically accumulated Li was lost, especially if depuration kinetics were rapid, three distinct rinsing protocols were tested. For this, 15 mussels were exposed for 1 h to seawater enriched with Li at a concentration of 1.5 mg Li/L, while five mussels were kept in natural seawater (0.18 mg Li/L) as a control group (“M-N” group).

After exposure, the contaminated mussels were randomly divided into three groups of five individuals each. In the first group (“M-E”), soft tissues were removed directly and freeze-dried without rinsing. In the second group (‘M-EKR3′), mussels were euthanized, and their soft tissues were held with forceps, dipped, and rinsed three times in natural seawater before freeze-drying. The third group (M-ELD30) consisted of live mussels transferred to natural seawater (0.18 mg Li/L) for 30 min, allowing their shells to open naturally before soft tissues were removed and freeze-dried. Control samples (M-N) were collected simultaneously, and their soft tissues were immediately freeze-dried for comparison.

### Li and Li Isotope Measurements

A detailed description of the analytical methods used in this study is available in refs^50^ and^56^. Briefly, all sample preparations were conducted in a positively pressurized clean laboratory under a fume hood, using only distilled, trace-metal-grade reagents and precleaned vessels to minimize contamination and procedural blanks. Mussel samples (0.3–0.4 g) were weighed into Teflon reactors and digested by microwave irradiation in a mixture of 5 mL of HNO_3_ and 2 mL of H_2_O_2_ (both trace metal grade, Fisher Scientific) at 90 °C for approximately 5 h. The resulting solutions were transferred to precleaned polyethylene tubes and gravimetrically diluted to 50 g with Milli-Q water (18 MΩ/cm). Each digestion batch included two procedural blanks and two replicates of the Certified Reference Material (CRM) IAEA-407 (Fish Homogenate, IAEA, Vienna), with CRM recoveries of 95 ± 3%. For each sample, two aliquots were separated for elemental and isotopic analyses.

Lithium concentrations were measured at the International Atomic Energy Agency (IAEA) in Monaco using flame atomic absorption spectroscopy (ContrAA 700, Analytik Jena, Germany). The biological reference material IAEA-407 was analyzed repeatedly during each session to ensure accuracy and reproducibility, achieving an external precision of better than 3% over seven repeated measurements. All potential sources of uncertainty were systematically identified, and combined standard uncertainties were calculated using the numerical differentiation method described in ref 57.

For Li isotope analysis, Li purification and separation were performed using AG 50-X12 cationic resin (200–400 mesh), following the method outlined in refs^56^ and^58^, with 1 N HCl as the eluent. Each batch of 20 columns included one blank and one in-house standard reference (Seawater 1-G). All procedures were carried out in a clean room at the Laboratory of Oceanography of Villefranche (LOV, CNRS and Sorbonne University). Most isotopic analyses were conducted at the CNRS-INSU National Service at the École Normale Supérieure de Lyon (France) using a Thermo Fisher Neptune *Plus* multicollector inductively coupled plasma mass spectrometer. Some of the samples were also analyzed using the Neoma MC-ICP-MS, following the procedure described in ref^55^. The accuracy of the method was validated by standard addition to fish muscle reference material,^56^ and the Li isotope value of the standard seawater solution (SW-1G: EDMM-1-G, Analytical Environmental Laboratory) was determined to be 31.8 ± 0.8‰ (n = 4, 2SD), using the Neptune *Plus*, and 31.21 ± 0.6‰ (n = 14, 2SD) for (SW-1H: EDMM-1-H) using the Neoma.

### Modeling Li Uptake and Excretion

To understand how mussels accumulate and release Li over time, we developed a parameterized box model. This model simulates the exchange of Li between mussels and their surrounding seawater across three distinct phases:

Initial control phase, where mussels were exposed to baseline Li concentrations.
Bioaccumulation or exposure phase, where mussels were exposed to elevated Li concentrations in seawater.
The depuration phase, where mussels were returned to baseline Li conditions to observe how they released accumulated Li.

The model is close to the one developed in refs 59–61 but adapted to our experimental setup, with no particulate phase, and including Li isotopes. It is structured around three compartments with Li fluxes between them (see Figure 1): (a) seawater (SW), which acts as the source and as a waste of Li for the mussels (assumed to have an infinite Li mass, due to its large volume), (b) mussel soft tissues (MSL), where Li accumulates, and (c) the waste (WST), which represents what is excreted by mussels and diluted into the aquarium seawater. Detailed description of the modeling can be found in the Supporting Information. Key variables in the model include:

- m_SW_: mass of Li (ng) in seawater (set to infinite).
- m = m_MSL_: mass of Li (ng) in mussel (swept variable).
- m_WST_: mass of Li (ng) in waste (set to infinite).
- F_IN_ = F_SW‑MSL_: Li flux (ng/h) from seawater to mussel.
- F_OUT_ = F_MSL‑WST_: Li flux (ng/h) from mussel to waste.
- α_IN_ = α_SW‑MSL_: Li isotope fractionation factor corresponding to the Li input, with α_SW‑MSL_ = (^7^Li/^6^Li)_SW_/(^7^Li/^6^Li)_MSL_, ie. the deviation of Li isotope ratio caused by its uptake by mussels
- α_OUT_ = α_MSL‑WST_: Li isotope fractionation factor corresponding to Li excretion (from mussel back to seawater), with α_MSL‑SW_ = (^7^Li/^6^Li)_MSL_/(^7^Li/^6^Li)_SW_

**Figure 1:**
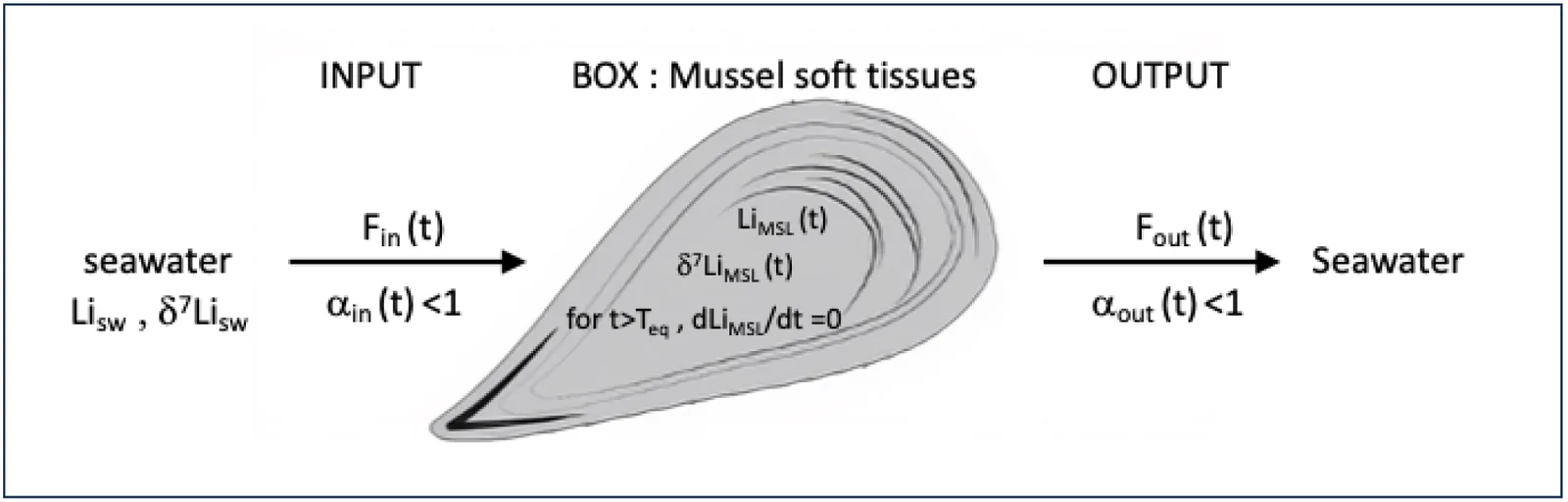
Box model of the Li mass and Li isotopes in the mussel soft tissue. Lithium isotopes ratios are reported in δ^7^Li, i.e., normalized to the LSVEC standard. Subscripts are MSL, mussel; SW seawater; IN, input; and OUT, output. For parameters definition, see text and Supporting Information.

#### Exposure Phase: How Mussels Absorb Li

During the exposure phase, the mass of Li in the mussel (m = m_MSL_) changes over time, starting from an initial value (m_t=0_) and approaching a target value (m_t=f_) as the mussel reaches equilibrium with the elevated Li concentration in seawater (at time t_eq_). This process is modeled using a quadratic function, derived from mass conservation differential equations for Li and Li isotopes,^22,62^ which ensures that

- The mass of Li in the mussel matches the initial and final range of values observed experimentally.
- The net change in Li mass becomes zero once equilibrium is reached (i.e., the mussel stops accumulating Li).

The time-dependent mass of Li in the mussel is described as follows:

- Before exposure (*t* < 0), the Li mass remains constant at its initial value (m = m_t=0_).
- During exposure (*t* ≥ 0 and < t_eq_), the Li mass follows a quadratic trajectory, m = (a/2)·t^2^+ b·t + c, where t_eq_is the time required for the mussel to reach equilibrium.
- After equilibrium (t ≥ t_eq_), the Li mass stabilizes at its final value (m = m_t=f_).

#### Depuration Phase: How Mussels Release Li

The depuration phase was modeled using the same principles as the uptake phase, but applied to the elimination of Li from the mussels placed in noncontamination conditions (seawater with baseline Li concentration). The same quadratic structure and equilibration time (t_eq_) were used with the additional constraint that the excretion rate (F_OUT_) remained continuous as the mussel transitioned from exposure to depuration. By combining the control, uptake, and depuration phases, we generated a complete time series of Li mass and fluxes for the entire experiment.

#### Li Fluxes, Isotopic Fractionation, and Model Scenarios

At each time step in the model, we assigned specific values to the Li masses in seawater (m_SW_) and in mussel tissues (m_MSL_). We also specified the isotopic composition of Li in seawater (δ^7^Li_SW_) since during control and depuration phases, δ^7^Li_SW_ = 31.2‰, while during the exposure phase, δ^7^Li_SW_ = 22.1‰ because of the Li enrichment using a Li salt that was slightly enriched in ^6^Li.

The isotopic fractionation factor for Li excretion (α_OUT_) was allowed to vary smoothly, reflecting biological adjustments in the mussel’s excretion process. This variation is expressed as 1000·ln­(α_out_) = Δ_out_, in ‰ and it changes in proportion to the relative rate of Li excretion. In contrast, the isotopic fractionation for Li uptake (α_in_) was assumed to change abruptly, mirroring the sudden change in Li influx when mussels are exposed to Li-rich seawater.

The flux data generated by the model were then organized into worksheets using the ISOBXR_MASTER workbook (part of the isobxr package; Tacail, 2021). A companion file, SCENARIO_MASTER, was used to define the sequence of model runs, the time steps, and the δ^7^Li values for seawater. Each scenario was simulated using the sim.scenario function in the isobxr package.

## Results

### Rinsing Tests

As illustrated in Figure 2, mussels *(Mytilus edulis)* exposed to Li-enriched seawater (Li_SW_ = 1.5 mg/L) exhibit significantly higher Li concentrations in their soft tissues compared to the control group (M-N), which was exposed to natural seawater (with a baseline Li concentration of 0.18 mg/L). Specifically, the average Li concentration in the control group is 0.87 μg/g, whereas mussels exposed to elevated Li levels show concentrations ranging from 3.8 to 8.8 μg/g. This indicates that uptake of Li in mussels is both rapid and efficient for all mussels.

**Figure 2:**
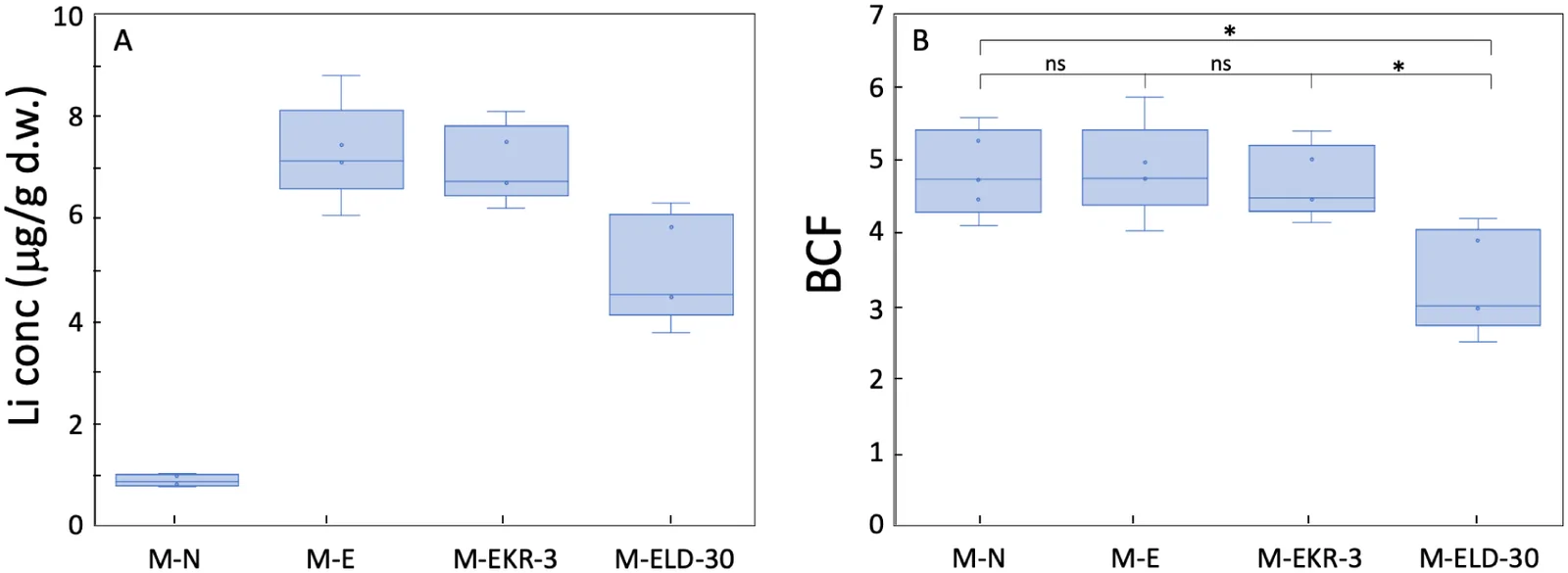
(A, B) Li concentrations in mussel soft tissues and related Li bioconcentration factors (BCF), corresponding to the rinsing tests. Each group includes 5 specimens. M-N represents mussels exposed to natural seawater (0.18 mg Li/L) for 1 h, then immediately freeze-dried; M-E represents mussels exposed to 1.5 mg Li/L for 1 h and then immediately freeze-dried; M-EKR3 represents mussels exposed to 1.5 mg Li/L for 1 h, tissues immediately removed, rinsed three times quickly in seawater, and freeze-dried; M-ELD30 denotes mussels exposed to 1.5 mg Li/L for 1 h and subsequently transferred as living specimens to natural seawater for 30 min (until shell opening), before tissue removal and freeze-dried. No significant difference among mussel weights is observed. Asterisks show the significance of the difference inferred through a Mann–Whitney–Wilcoxon tests: ns (not significant) for *p* > 0.05, * for *p* < 0.05.

An interesting observation arises when comparing mussels subjected to different postexposure (rinsing) treatments. Mussels that were kept alive for 30 min in control seawater after the exposure phase (i.e., the M-ELD30 group) display a significantly lower average Li concentration (4.99 ± 1.06 μg/g) compared to mussels that were either not rinsed (M-E group: 7.32 ± 0.99 μg/g) or rinsed three times quickly (M-EKR3 group: 7.06 ± 0.76 μg/g). However, the M-ELD30 mussels lost less than 30% of their accumulated Li on average, suggesting that while some Li was released, a substantial portion remained within their tissues.

The bioconcentration factors (BCF), defined as the ratio of Li concentration in soft tissues to that in the surrounding water, reveal no statistically significant differences between the control group (M-N: BCF = 4.84 ± 0.6, n = 5), unrinsed mussels (M-E: BCF = 4.71 ± 0.50, n = 5), and mussels rinsed three times quickly (M-EKR3: BCF = 4.88 ± 0.66, n = 5). This consistency in BCF values across these groups suggests several key points.

First, the rinsing process with natural seawater does not significantly alter the Li concentration in mussel tissues. This implies that this rinsing method effectively removes only surface-adsorbed Li without affecting the Li that has been absorbed into the soft tissues. This aligns with established protocols, where quick rinsing is commonly used to eliminate adsorbed metals, particularly those that are loosely bound.^63^ We also conclude that the amount of Li adsorbed on the mussel surface is negligible compared to the Li taken up internally, in agreement with the known weak adsorption capacity of this alkali monovalent ion.^58^

Despite the overall consistency in Bioconcentration Factor (BCF) values among most groups, the mussels subjected to a 30 min rinse while still alive (M-ELD30 group) exhibit a statistically significantly lower BCF (3.32 ± 0.71, n = 5), by 30% compared to the other three groups (Figure 2B). This suggests that Li depuration in*Mytilus edulis* is an active excretion mechanism. The fact that depuration begins shortly after the end of the exposure phase—even within a brief 30 min window—highlights the efficiency of this biological response and suggests that *Mytilus edulis* actively regulates Li levels in its tissues. The good reproducibility and the absence of differences in mussel dry weight further support that the observed decrease in BCF in group M-ELD30 is due to biological processes rather than physiological or methodological artifacts.

### Exposure Phase

In this second experiment, mussels were abruptly exposed to artificially Li-enriched seawater (1.5 mg Li/L) for durations ranging from 0.5 to 5 days, while the control group remained in natural seawater (containing 0.18 mg Li/L).

Over the whole course of the study, control mussels maintained a consistent Li concentration of 0.85 ± 0.08 μg/g dry weight (d.w.) (n = 24, SD) (see Table 1 and Supporting Information) (SD = 1x standard deviation). In contrast, mussels exposed to Li-enriched seawater exhibited significantly higher Li concentrations, ranging from 1.5 to 3.8 μg/g d.w., with an average value of 2.5 ± 0.6 μg/g d.w. (n = 24, SD), demonstrating clear bioaccumulation under excess dissolved Li. This difference in tissue Li levels is further reflected in the BCF, which we can also estimate in control conditions, since natural seawater contains some Li (0.18 mg/L). Control mussels exhibit BCF values ranging from 3.8 to 5.7, with an average of 4.7 ± 0.4 (n = 24, SD), whereas Li-exposed mussels display markedly lower BCFs, ranging from 0.8 to 2.3, with an average of 1.5 ± 0.4 (n = 24, SD).

**Table 1:** Data Summary (Average ± 2SE) for the Exposure and the Depuration Phases (See Also Supporting Information)

	**Exposure phase (0–5 days)**					**Depuration phase (5 – 15 days)**					
	BCF	Δ^7^Li_MSL‑SW_ ‰	[Li]_MSL_ μg/g d.w.	Li_MSL_ tot μg Li	δ^7^LiMSL ‰	BCF	Li_depurated_ %	Δ^7^Li_MSL‑SW_ ‰	[Li]_MSL_ μg/g d.w.	Li_MSL_ tot μg Li	δ^7^Li_MSL_ ‰
**CTRL**	4.7 ± 0.4	0.5 ± 0.6	0.9 ± 0.1	0.8 ± 0.1	31.9 ± 0.6	4.8 ± 0.8	n. a.	0.3 ± 0.4‰	0.9 ± 0.2	0.8 ± 0.1	32.0 ± 0.3
**Time #1** ^a^	1.9 ± 0.4	7.4 ± 0.3	3.0 ± 0.6	2.1 ± 0.1	29.6 ± 0.3	5.2 ± 0.7	73% ± 3‰	0.4 ± 1.7‰	0.9 ± 0.1	0.7 ± 0.1	31.7 ± 1.6
**Final time** ^b^	1.9 ± 0.3	6.3 ± 0.8	3.1 ± 0.5	2.5 ± 0.2	27.9 ± 0.8	5.5 ± 0.9	72% ± 7‰	1.3 ± 0.3‰	1.0 ± 0.2	0.7 ± 0.2	32.6 ± 0.3

a 12 h after onset of exposure in SW with Li = 1.5 mg/L and δ^7^Li = 22.1‰; 6 h after onset of depuration in a control SW (Li = 0.18 mg/L and δ^7^Li = 31.3‰).

b 5 days after onset of exposure; 10 days after onset of depuration.

Thus, despite the dissolved Li concentration being 9 times higher than in the control aquarium, the BCF values are 3 times lower than under control conditions, suggesting that mussels accumulate proportionally less Li when exposed to higher environmental concentrations.

Li isotopes were also analyzed for these samples. First, compared to the control seawater δ^7^ Li value of 31.2 ± 0.4‰ (n = 5, SD), the Li-enriched seawater has a distinct δ^7^ Li of 22.1 ± 0.3‰ (n = 5, SD) that reflects the isotopic composition of the Li standard used for enriching the seawater.

As a consequence, it is not possible to directly compare the δ^7^ Li values of mussels from different groups. In order to facilitate this comparison, we use the Li isotopic fractionation expressed as Δ^7^Li_msl‑sw_ = δ^7^Li_msl_ - δ^7^Li_sw_, which represents the difference in δ^7^Li value between mussels and their aquarium seawater. In control conditions Δ^7^Li_msl‑sw_ range from −0.4‰ to +0.8‰ with an average of 0.4 ± 0.5‰ (n = 12, SD) indicating that control mussels display the same δ^7^Li as their surrounding seawater, within uncertainties. In contrast, in Li-rich aquarium, the mussel-water isotopic fractionation Δ^7^Li_msl‑sw_ are significantly larger, ranging from +5.4‰ to +10.4‰, with an average of +8.2 ± 1.3‰ (n = 24, SD). These mussels therefore display a significant enrichment in the heavier isotope (^7^Li) relative to both control mussels and exposure seawater (see Figure 3).

**Figure 3:**
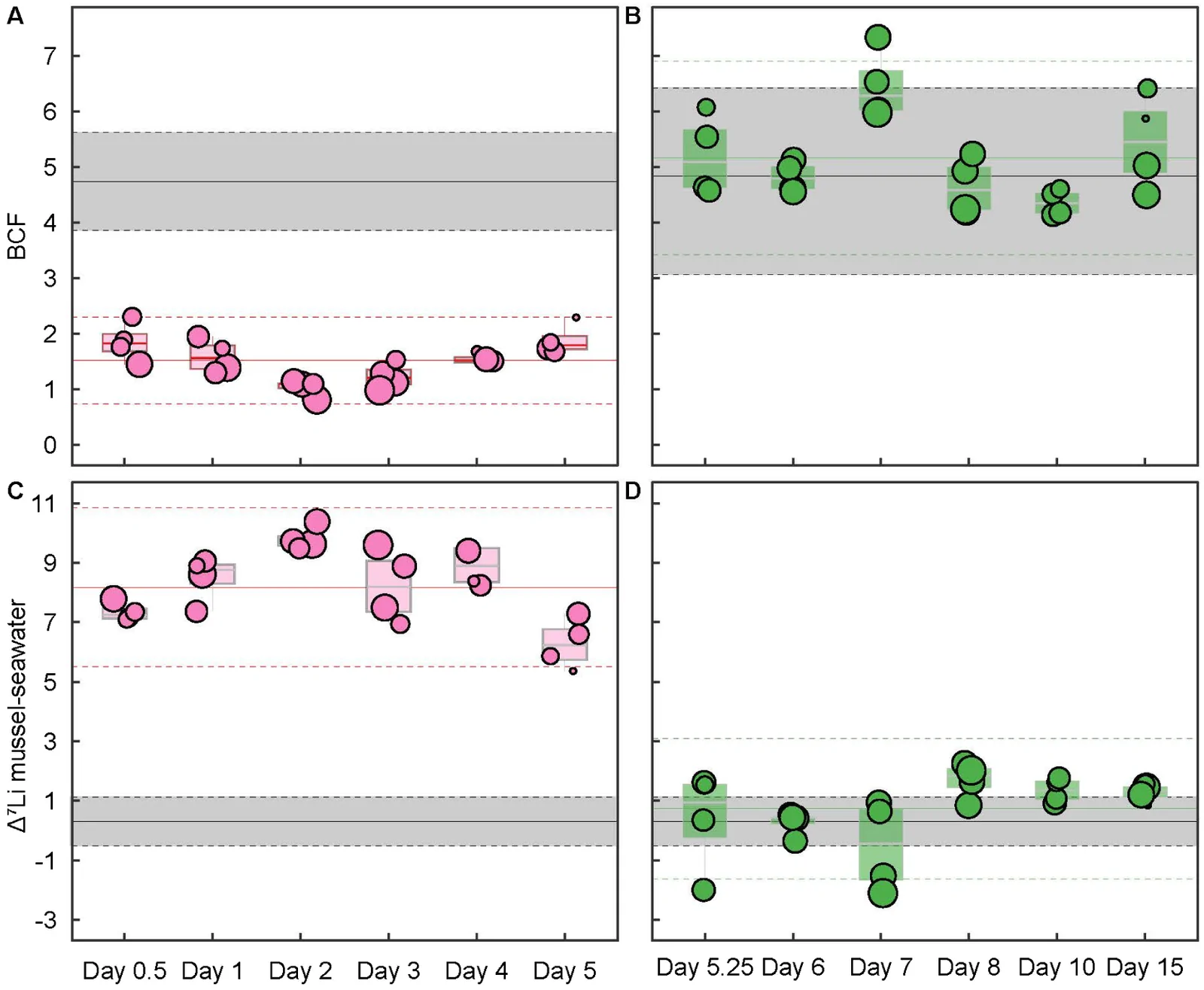
(A, C) Bioconcentration Factor (BCF) and Δ^7^Li_mussel‑seawater_ values for*Mytilus edulis*exposed to Li-rich seawater (exposure experiment, in pink) as a function of exposure time. The gray-shaded areas represent the average BCF (4.7 ± 0.9, *n* = 24, 2SD) and Δ^7^Li_msl‑sw_ (0.3 ± 0.8‰, *n* = 6, 2SD) for the control group monitored over the same time period. Symbol size is proportional to mussel dry tissue weight (same as in Figure 4). (B, D) Bioconcentration Factor (BCF) and Δ^7^Li_msl‑sw_ values for*Mytilus edulis*submitted to depuration in natural seawater for a time ranging between 0.25 and 10 days (in green) after their exposure. The gray-shaded regions represent the average BCF (4.8 ± 0.8, *n* = 24, 2SD) and Δ^7^Li_msl‑sw_ (0.4 ± 1‰, *n* = 12, 2SD) values for the control group monitored over the same time period (see Supporting Information).

These results align well with previously published exposure experiments for the same species under similar Li enrichment conditions,^22^ confirming the reliability and reproducibility of our experimental and analytical approach. It should be noted that, while Thibon et al.^22^ exposed mussels for a fixed 4-day period, the present study includes multiple sampling time points from 0.5 to 5 days. Taken altogether, the data from these two exposure studies show that BCF and Δ^7^Li_msl‑sw_ display a negative correlation (shown in Figure 4). As shown in Figure 3, both parameters also antivary as a function of time over the exposure duration. These trends suggest that both parameters are controlled by the same factor(s) likely related to interspecimen differences.

**Figure 4:**
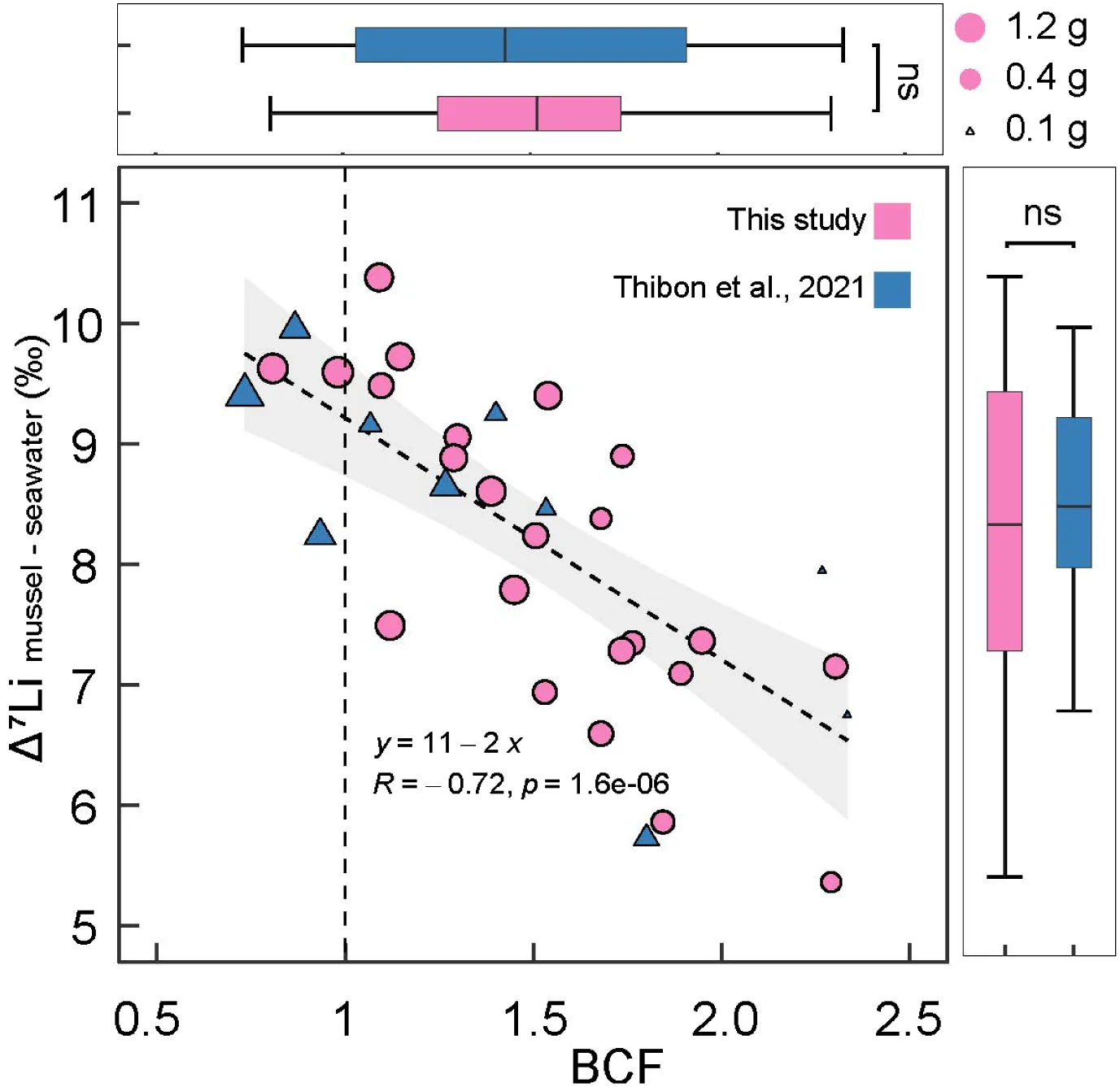
BCF versus Δ^7^Li_msl‑sew_ for*Mytilus edulis*exposed to Li-rich seawater (1.5 mg Li/L) only, from this study (in pink) and from ref^22^ (in blue). Pink circles represent the average value for 3 specimens (pooled); the blue triangle represents one individual mussel. Symbol sizes are proportional to mussel dry tissue weights (normalized).

### Depuration Phase

After 5 days of exposure to 1.5 mg Li/L seawater during the first phase, mussel specimens were transferred to an aquarium containing natural seawater (0.18 mg Li/L) for periods ranging from 6 h (0.25 day) to 10 days (see Figure 3B,D) in the aim of monitoring the Li depuration process.

Li concentrations measured in the soft tissues of mussels sampled over this period of time are consistently low, averaging 0.93 ± 0.16 μg/g (n = 24, SD), with values spanning from 0.74 to 1.32 μg/g. These concentrations are close to those measured in control mussels, either collected simultaneously (0.87 ± 0.15 μg/g, n = 24), or collected previously during the exposure experiment (0.84 ± 0.08 μg/g, n = 24). Notably, within the first 6 h of depuration (by day 5.25), Li concentrations in the soft tissues drop rapidly from 2.5 ± 0.57 μg/g (n = 24, SD) to 0.94 ± 0.13 μg/g (n = 4, SD). In mussels submitted to depuration into natural seawater (0.18 mg Li/L), BCF values range from 4.14 to 7.33, with a mean BCF of 5.16 ± 0.73 (n = 24, SD) (BCF values are calculated using the natural seawater Li concentration). As depuration progresses, no statistically significant differences are observed between the BCF values of the experimental group and the control group (4.84 ± 0.42, n = 24, SD).

Concerning the isotopic fractionation, Δ^7^Li_msl‑sw_ varies within a narrow range during the depuration phase, from −2.1 to +2.3‰, averaging +0.7 ± 1.2‰ (n = 24, SD). This is close to that of the control group (0.3 ± 0.4‰, n = 6, SD). A rapid decrease in Δ^7^Li_msl‑sw_ values is observed, dropping from an average of +6.3 ± 0.8‰ (n = 24, SD) on day 5, at the end of the exposure period, to 0.4 ± 1.7‰ (n = 4, SD) by day 5.25, so after 6 h of depuration only.

### Modeling Results

For each Li-cycle simulation scenario, we compiled the model outputs, combined them with the corresponding parameter sets and run metadata, and retained only the simulations that fell within a two-standard-error (2SE) envelope of the experimental data (see Supporting Information). The model is underconstrained due to the rapid kinetics, as equilibrium was reached before the first sampling step (12 h during the exposure phase and 6 h during the depuration phase). However, fitting this equilibrium is achievable, enabling us to estimate the mean values and standard deviations for the targeted parameters listed in Table 2.

**Table 2:** Mean Value and Standard Deviation for Several Parameters Calculated by the Box Modeling Described in Figure 1 ^a^

Parameter	Mean	SD	Unit
^b^α_IN_/α_OUT initial_	1.0006	0.0003	
α_IN_/α_OUT eq_	1.008	0.001	
F_IN_(Li) initial	482	139	ng/h
F_IN_(Li) eq	1629	997	ng/h
F_OUT_(Li) initial	482	139	ng/h
F_OUT_(Li) eq	1629	997	ng/h
T eq	4.2	3.1	h

a At the initial time (t = 0) as well as after the time required to reach equilibrium (t_eq_), the input and output Li fluxes are equal.

b The isotopic fractionation factor between seawater and mussel (α_IN_ = (^7^Li/^6^Li)_SW_/(^7^Li/^6^Li)_MSL_)) is a simple function of Δ^7^Li_SW‑MSL_ that can be expressed as Δ^7^Li_SW‑MSL_ = 1000.ln­(α_IN_).

First, results show that the isotopic fractionation factors are consistent with those reported in published studies for active and passive transporters,^34^ as well as for similar Li exposure conditions of*Mytilus edulis*.^22^ Building on the exposure studies in Thibon et al.,^22^ and those performed here, we also investigate the depuration phase to provide additional constraints on both isotopic variations and output flux. This allows us to propose a more comprehensive and better constrained approach.

The modeling results indicate that individual uptake and output fluxes can range between 367 ng/h to 4073 ng/h, accounting for the variability observed in individual mussels of different weights. On average, when equilibrium is reached, both fluxes operate, respectively, at 1630 ng/h, reflecting a high capacity for Li transfer between seawater and the biological system, and vice versa.

## Discussion

### Biological and Environmental Controls of Mussel Li Mass

In this study, most mussels exposed to Li-enriched seawater for up to 5 days exhibit net Li bioaccumulation, as evidenced by higher Li concentrations and higher total Li content compared to control mussels (see Table 1 and Supporting Information). However, their bioconcentration factors (BCF) are lower than those of the control group. This suggests either an active excretion process during exposure, or a limitation of the Li uptake, even though the net flux remains in favor of Li bioaccumulation (e.g., total Li in exposed mussels/total Li in control mussels = 2.9 ± 0.5).

Following exposure, the depuration process is rapid: mussel Li concentrations and total Li content decrease toward control values in less than 6 h, while BCF values return to their initial levels.

Throughout the experiment, we also observe small but significant temporal variations in BCF values (Figure 3). Further analysis reveals that the mussel size is the primary factor explaining these apparent fluctuations. As shown in Figure 5, BCF exhibits a strong negative correlation with mussel dry weight that is consistent with published results: at any given exposure time, heavier mussels display lower Li concentrations.

**Figure 5:**
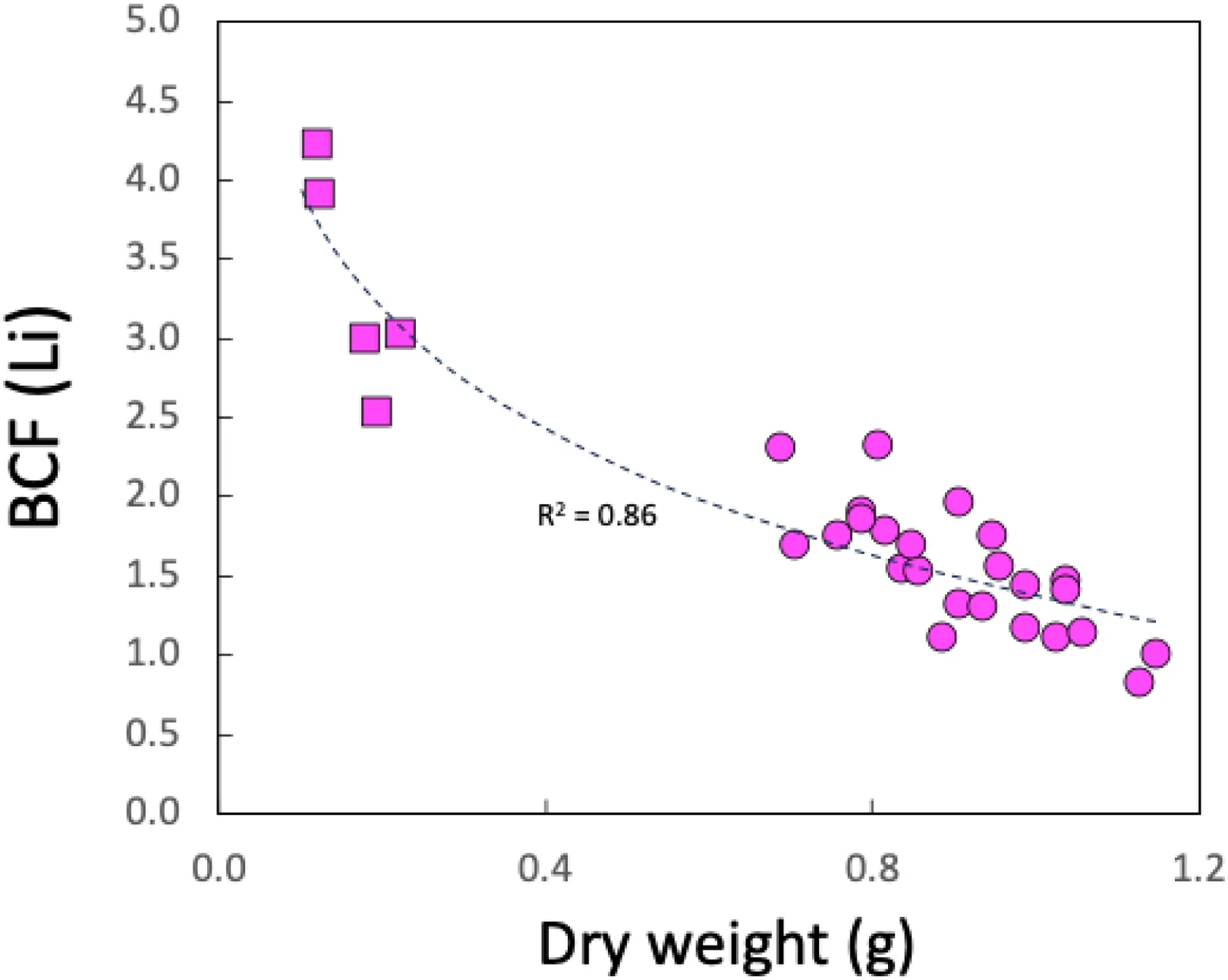
Li bioconcentration factor (BCF­(Li)) versus mussel dry tissue weight for*Mytilus edulis*exposed to Li-rich seawater (1.5 mg Li/L). Data from the bioaccumulation period (pink circles) and from the rinse tests (pink squares). All samples were rinsed alive for 30 min.

Several studies have demonstrated that smaller or lighter mussels typically exhibit higher metal concentrations in their tissues compared to larger individuals.^58–66^ For example, concentrations of metals or trace elements such as As, Cd, Co, Cr, Cu, Fe, Mn, Ni, Pb, and Zn in the soft tissues of*Mytilus edulis*were found to be inversely correlated with tissue dry weight.^67–71^ This relationship was best described by power functions, where metal concentration decreases as a power of tissue dry weight.^65,67^

The inverse correlation shown in Figure 5 could be attributed to a dilution effect during mussel growth. As highlighted by^64^ and recently confirmed in various marine species,^12,52^ Li tends to accumulate primarily in specific organs, such as the kidneys (2.7 μg/g in mussels), compared to other tissues like gills (0.76 μg/g), mantle (0.27 μg/g), or foot (0.28 μg/g). This uneven distribution was also observed in scallops, where higher Li concentrations are found in the digestive gland (0.76 μg/g) and gills (0.60 μg/g), while lower concentrations are detected in muscle (0.09 μg/g) and gonad (0.29 μg/g). When analyzing bulk tissue—as in this study—the proportion of high-Li organs, such as kidneys, is lower in larger mussels than in smaller ones, resulting in a smaller Li concentration in bulk (total) tissue.

There is also a strong anticorrelation between BCF and Δ^7^Li (isotopic fractionation), which suggests that Li homeostasis also varies with mussel weight since isotopic fractionation essentially results from active Li biological transfer.^34^ Indeed, a simple dilution process would not be expected to alter isotopic composition because first there is not a large difference between δ^7^Li among organs and tissues in bivalves, and second, bivalve muscles contain very little Li amounts (<0.1 μg).^12^ Differences in mussel δ^7^Li values therefore imply that either the absorption (input) or excretion (output) fluxes—or both—must differ between small and large mussels. This hypothesis is explored further in the following section.

Additional insights can be drawn from a compilation of literature data. Figure 6 compares results from previous publications reporting Li concentrations in mussel tissues with those in corresponding exposure waters.^20–23,72^ Each data set exhibits a strong linear relationship between water and soft tissue Li concentrations, indicating that Li bioaccumulation in mussels is largely driven by ambient water Li levels. However, the regression slopes vary significantly among the studies. Notably, the freshwater mussel *Dreissena polymorpha* ^72^ exhibits the steepest slope compared to marine species such as*Mytilus edulis* ^22^ and*Mytilus galloprovincialis*.^20,21,23^ This difference is not explained by variations in size or weight (unshown here in detail). For instance, *Mytilus galloprovincialis* mussels studied by Fraga et al.^21^ are approximately half the length of those analyzed by Cunha et al.,^23^ yet their regression slopes for Li bioaccumulation are remarkably similar.

**Figure 6:**
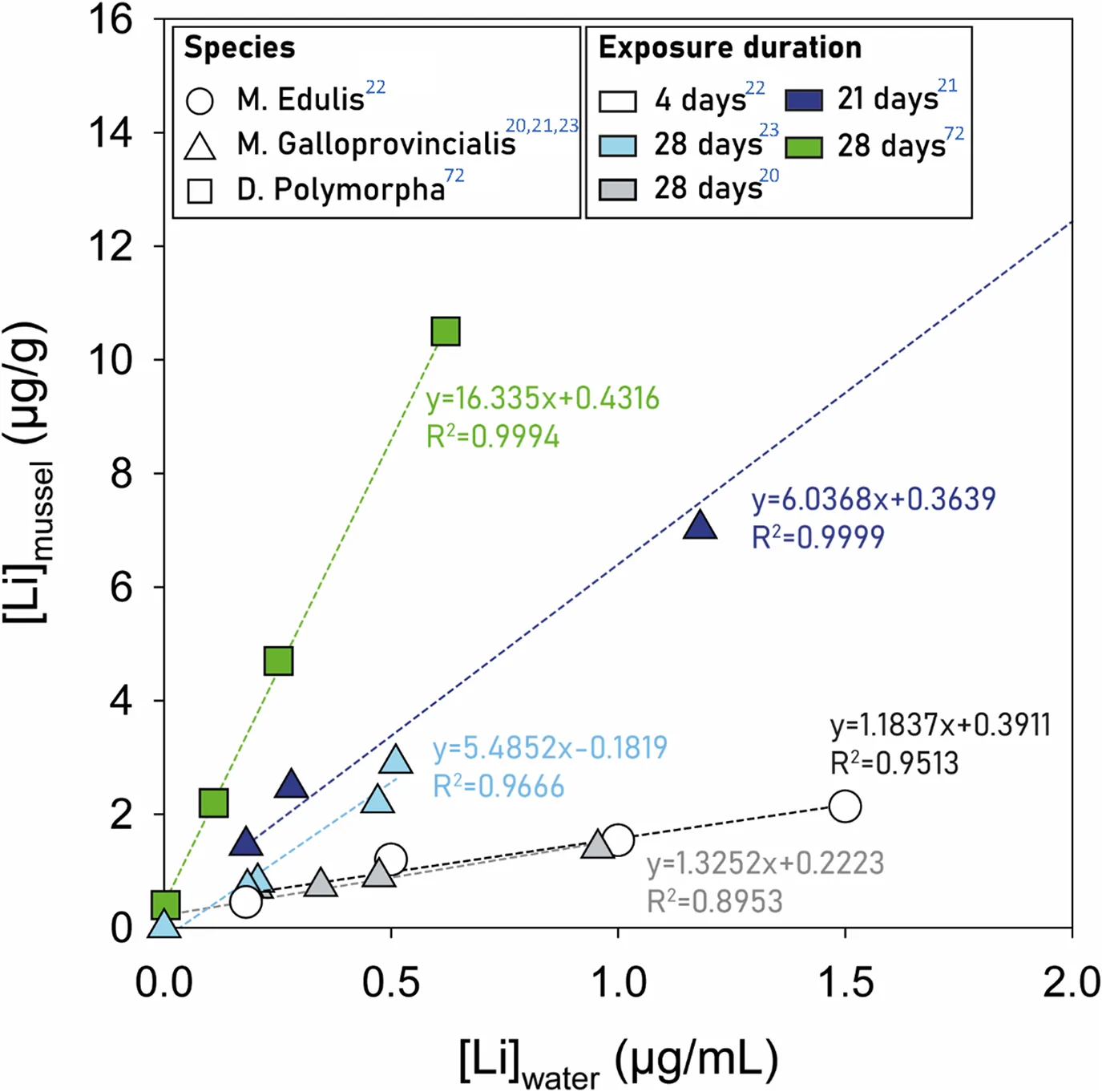
Relationships between Li concentrations in mussel soft tissue (μg/g) and in water (μg/mL), as compiled from previous laboratory studies. Symbols represent species *(Mytilus edulis*, circles; *Mytilus galloprovincialis*, triangles; *Dreissena polymorpha*, squares), and colors indicate exposure duration.^20–23,72^ Green squares represent freshwater mussels, while all other colors correspond to seawater ones.

The various trends compiled in Figure 6 strongly suggest that Li bioaccumulation is significantly greater in low-salinity environments compared to saline conditions. The variability in the slopes can be attributed to the combined influence of physiological and environmental factors. The fact that the chemical composition of the exposure medium plays a critical role can be explained by known behavior of Li transporters present in all living species. Indeed, due to the chemical similarity between Li^+^ and Na^+^, Li^+^ can exploit shared ion transport pathways.^22,34,72–74^ In freshwater, low dissolved sodium concentrations (compared to saline environment) reduce Li–Na competition, potentially enhancing Li^+^ uptake through Na^+^ transport systems and therefore bioaccumulation.^34,72^

An alternative explanation may be related to the kidney functioning. Although gills also participate in ion exchange, the kidney remains the primary organ for waste elimination and sodium regulation, ensuring internal stability amid fluctuating salinity. Marine and freshwater bivalves possess specialized excretory organs (nephridia) that filter hemolymph to produce urine. To maintain osmotic balance, their kidneys, connected to the pericardial cavity, perform filtration, selective reabsorption (e.g., water, sodium, and glucose), and active secretion. For freshwater mussels, a significant amount of Na filtered from hemolymph is partially reabsorbed in the ureter—preventing excessive ion loss. This reabsorption is less critical for osmoregulation in marine mussels, since they live in Na-rich waters. As a consequence, this process may explain the dichotomy observed between freshwater and marine mussels shown in Figure 6. Additionally, interspecific and genetic differences in metabolic activity may influence the rate at which equilibrium is reached and the efficiency of Li retention in soft tissues.

### Evolution and Controls of Li Fluxes

The kinetics of Li uptake and depuration proved to be more rapid than anticipated. As a result, we were unable to capture the early evolution of Li concentrations and δ^7^Li values within the first 12 h of bioaccumulation and the first 6 h of the depuration process. Nevertheless, we can propose simulations based on concentration and isotopic data and on the current knowledge of biological isotopic fractionation, e.g., ref^34^, in order to provide orders of magnitude for input and output Li fluxes, as presented in Table 2, Figure 7, and in the Supporting Information.

**Figure 7:**
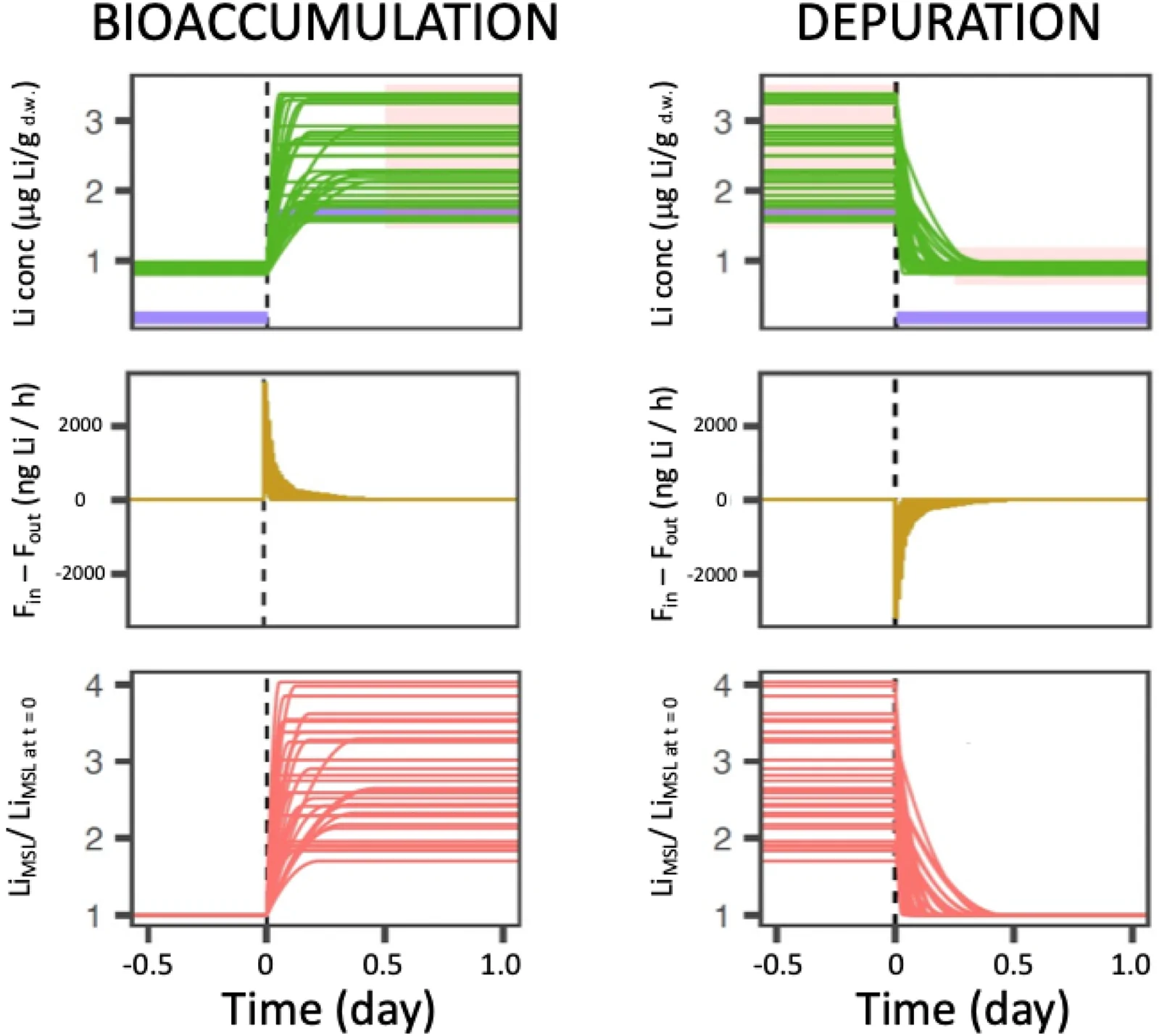
Selected results for the modeling described in the Li and Isotope Measurements section and in the Supporting Information.

Although the model remains underconstrained, the coupling of Li concentrations to Li isotopes reveals several key insights. Specifically, the δ^7^Li values and the mass of Li in mussels during the exposure phase can only be explained if the ratio of isotopic fractionation factors (α_in_/α_out_) is 1.008 ± 0.001. This implies that under Li-rich conditions, the isotopic fractionation factor associated with the excretion process becomes larger in magnitude compared to the input flux. In contrast, before the exposure experiment, at time t = 0 (initial time), the ratio (α_in_/α_out_) is significantly different, closer to 1, at a value of 1.0006 ± 0.0003. Under control conditions, isotopic fractionation factors corresponding to uptake and excretion are similar within uncertainties.

This observation of larger isotopic fractionation related to the excretion process aligns with the role of Na–H exchangers, regulating salt levels and excretion in the kidneys. Indeed, as described above, it has been shown by Poet et al.^34^ that both Na^+^ and Li^+^ are transported through the same exchangers (e.g., NHEs), and this transport mechanism results in significant Li isotopic fractionation. Interestingly, their paper shows that a cooperative effect operates at the cellular level, meaning that when the Li concentration of the external medium is higher, the magnitude of Li isotopic fractionation increases. This effect has been shown to be a hallmark of a dimeric transport by NHE proteins causing cooperativity of ^6^Li transport when the external Li concentrations increase.^34^ It is therefore consistent with findings that more Li in seawater leads to larger biological isotopic fractionation.

In contrast to the initial isotopic modeling proposed by Thibon,^22^ our experiment also enables us to quantify the depuration flux and its temporal evolution. Average Li output (excretion) flux ranges between 482 and 1629 ng/h, rapidly increasing during the accumulation phase and decreasing during the depuration phase. The time required to reach equilibrium during the exposure phase is estimated at 4.2 ± 3.1 h, significantly shorter than our first sampling time at 12 h. This rapid equilibrium is consistent with our rinsing tests described in the Materials and Methods section, which show that living mussels lose approximately 30% of their Li within 30 min. It also aligns with the depuration phase, where about 72% of the mussel’s Li content is lost in less than 6 h (Table 1).

Figure 7 illustrates that, during the initial hours of exposure, the net flux (F_IN_– F_OUT_) is positive due to a high F_IN_ (ranging from 200 to 4000 ng/h). However, this net flux rapidly decreases as F_OUT_ increases. After approximately 4 h, equilibrium is reached, at which point F_IN_ and F_OUT_ become equal in magnitude. In contrast, during the first 6 h of the depuration phase, mussels rapidly lose Li because the input flux (F_IN_) decreases abruptly. Initially, F_OUT_ dominates but equilibrium is quickly reestablished as F_OUT_ diminishes over time. At equilibrium, F_IN_ once again equals F_OUT_.

The substantial Li fluxes observed at equilibrium in our experiments indicate that mussels used for biomonitoring would primarily capture short-term environmental variations in Li concentrations, as they may rapidly lose most of their bioaccumulated Li. However, our compilation in Figure 6 demonstrates that culture duration does not significantly affect the bioaccumulation capacity of mussels, at least for the marine species studied yet. Indeed, similar results are observed for 4-day and 28-day exposures. In addition, the excellent regression coefficients in Figure 6, including at short exposure durations, suggest that the water Li concentration may play a limited role in the Li transport kinetics. These findings, however, raise new questions concerning bivalve Li retention time and suggest that further experiments are necessary before mussels can be reliably employed as sentinel species for Li contamination.

## Conclusions

Mussels are key indicators or “sentinel” organisms in coastal ecosystems, and through the study of Li isotopes in their soft tissues, we can gain an in-depth understanding of the levels and biogeochemical processes of Li in littoral environments. The rapid kinetics of Li uptake and release observed in this study suggest that mussels can effectively capture short-term variations in environmental Li concentrations. They rapidly reach equilibrium and the culture duration does not affect their chemical composition. This also raises new questions concerning their use as sentinel species for monitoring Li contamination over the long-term.

The significant isotopic fractionations that we observed during exposure evidence the great sensitivity of Li isotopes to trace changes in biological activity related to environmental contamination and offer a new perspective for detecting potential toxic effects.

Another key finding of this study is the negative correlation between bioconcentration factors (BCFs) and mussel dry weight, suggesting that larger mussels accumulate less Li per unit of weight. This relationship underscores the importance of considering the mussel size or weight in biomonitoring programs. Additionally, this study highlights the need for standardized protocols in rinsing mussels before measurements in order to avoid active excretion of Li, which may lead to an underestimation of bioaccumulation levels.

Overall, this study contributes to our understanding of Li dynamics in marine ecosystems and emphasizes the need for further research to elucidate the early kinetics of Li bioaccumulation and depuration. The findings have implications for the use of mussels in biomonitoring programs and for assessing the ecological risks associated with increasing levels of Li contamination in coastal environments.

## Supplementary Material





## Notes

Li concentration and Li isotopic composition of mussels during the exposure and the depuration phases (XLSX)
Modeling bioaccumulation and depuration: parameter space definition and sampling; : parameter ranges used in the sweep (Table S1); descriptive statistics used for fitting simulations (Table S2); example of modeled elemental and isotopic forcings (Figure S1); and modeling results for a selection of parameters (Figure S2) (PDF)
