# Supplementary material for: High Lithium Bioaccumulation and Depuration Rates in Marine Mussels (Mytilus edulis): An Isotopic Perspective
Source: ACS Omega. 2026 Oct 22;11(43):66386–98. doi: 10.1021/acsomega.6c05538 (PMC13737927; doi:10.1021/acsomega.6c05538)
Supplement: Supplementary file 1 [file ao6c05538_si_002.pdf]

High lithium bioaccumulation and depuration rates in marine mussels (*Mytilus edulis*): an isotopic perspective

Dongyu Chen[a,b], Théo Tacail[c], Yuki Mark[b], François Oberhänsli[b], Maryline Montanes[a], Hyeryeong Jeong[a], Emilia Vassileva[b], Anna Maria Orani[b], Laurine Payant[a], Arbia Jouini[a], Fanny Thibon[a], Philippe Telouk[d], Marc Métian[b], Nathalie Vigier[a,]*

[a] Laboratoire d'Océanographie de Villefranche-sur-Mer (LOV), CNRS, Sorbonne Université, 06230 Villefranche-sur-Mer, France

[b] IAEA Radioecology Laboratory, Department of Nuclear Sciences and Applications, 98000 Monaco, Principality of Monaco

[c] The Isotoparium, Division of Geological and Planetary Sciences, California Institute of Technology, Pasadena, CA 91125, United States

[d] Laboratoire de Géologie de Lyon, Ecole Normale Supérieure de Lyon, CNRS UMR 5276, Université de Lyon, 46 Allée d'Italie, 69007 Lyon, France

**corresponding author : nathalie.vigier@imev-mer.fr*

SUPPLEMENTARY INFORMATIONS

Content :

. Modeling bioaccumulation and depuration: parameter space definition and sampling

. Table S1: *Table of parameter ranges used in the sweep*

. Table S2: *Descriptive statistics used for fitting simulations*

. Figure S1: *Example of modeled elemental and isotopic forcings*

. Figure S2: *Modeling results for a selection of parameters.*

Modeling bioaccumulation and depuration: parameter space definition and sampling

A parametrized box model was developed, as detailed in the main text and in figure 1, taking into account Li input and output fluxes (F_{IN} and F_{OUT}) and respective isotopic fractionation factors (α_{IN} and α_{OUT}).

During the exposure phase, the instantaneous rate of change in Li mass (dm/dt) is derived from this trajectory:

- Before exposure ($t < 0$): There is no change in Li mass ($dm/dt = 0$).
- During exposure ($0 \le t \le t_{eq}$): The rate of change is given by $dm/dt = a \cdot t + b$, which represents the difference between the influx (F_{IN}) and outflow (F_{OUT}) of Li.
- After equilibrium ($t > t_{eq}$): The net change in Li mass is zero ($dm/dt = 0$), indicating that the mussel has reached a steady state where Li influx equals Li outflow.

We assumed that the inward flux of Li to mussels (F_{IN}) increases abruptly when the mussels are transferred from control condition to Li-rich seawater. The outward flux (F_{OUT}) is then defined as: $F_{OUT} = F_{IN,t=f} – a \cdot t + b$. This equation reflects the biological response of the mussel, where excretion gradually increases to match the new, higher influx of Li.
The model is solved step-by-step (with a time step of $dt = 0.2$ hours) until the mussel reached equilibrium, i.e. where F_{IN} equals F_{OUT}.

The instantaneous rate of change in Li mass (dm/dt) is derived from this trajectory:

- Before exposure ($t < 0$): There is no change in Li mass ($dm/dt = 0$).
- During exposure ($0 \le t \le t_{eq}$): The rate of change is given by $dm/dt = a \cdot t + b$, which represents the difference between the influx (F_{IN}) and outflow (F_{OUT}) of Li.
- After equilibrium ($t > t_{eq}$): The net change in Li mass is zero ($dm/dt = 0$), indicating that the mussel has reached a steady state where Li influx equals Li outflow.

We assumed that the inward flux of Li to mussels (F_{IN}) increases abruptly when the mussels are transferred from control condition to Li-rich seawater. The outward flux (F_{OUT}) is then defined as: $F_{OUT} = F_{IN,t=f} - a \cdot t + b$. This equation reflects the biological response of the mussel, where excretion gradually increases to match the new, higher influx of Li.
The model is solved step-by-step (with a time step of dt = 0.2 hours) until the mussel reached equilibrium, i.e. where F_{IN} equals F_{OUT}.

Quadratic parameterizationwas used to link model parameters to observations. For each model run, the coefficients of the quadratic function (a, b, and c) were calculated directly from the experimental data:

- $a = -2\,(m_{t=f} - m_{t=0}) / t_{eq}^2$: this coefficient determines the curvature of the Li mass trajectory
- $b = 2\,(m_{t=f} - m_{t=0}) / t_{eq}$: this coefficient determines the linear rate of change in Li mass
- $c = m_{t=0}$: this reflects the initial Li mass in mussels
- $F_{IN,t=f} = b + F_{IN,t=0}$: this defines the final influx rate of Li once equilibrium is reached.

These parameters allowed us to simulate the evolution of Li mass in the mussel, as well as the corresponding influx and outflow rates, across all phases of the experiment. Additional details about the parameter space, sampling methods, and simulation ranges are provided in the Supplementary Information.
We defined a multidimensional parameter space covering plausible combinations of kinetic and equilibrium Li isotope fractionation factors and associated mass-balance coefficients. Each free parameter was assigned lower and upper bounds and randomly swept across 100’000 combinations.
For each sampled combination, we computed two diagnostic ratios:

- Li isotope fractionation factors during exposure: $\alpha_{IN,t=f}$ / $\alpha_{OUT,t=f}$
- "Baseline" fractionation ratio: $\alpha_{IN,t=0}$ / $\alpha_{OUT,t=0}$

Only parameter sets satisfying physiologically and experimentally plausible constraints ($1.005 \leq \alpha_{IN,t=f} / \alpha_{OUT,t=f} \leq 1.011$ and $0.999 \leq \alpha_{IN,t=0} / \alpha_{OUT,t=0} \leq 1.002$) were retained. This filtered ensemble was saved as the input set for forward simulations of Li elemental and isotopic dynamics.

Table S1: Table of parameter ranges used in the sweep

**parameter**	**unit**	**Lower bound**	**Upper bound**
m_{SW}	ng	1.00E+15	
$m_{t=0}$	ng	600	1000
$m_{t=f}$	ng	1200	3200
$F_{IN,t=0}$	ng/h	100	700
t_{eq}	h	0.5	12.5
t_{exp}	h	5	
$\alpha_{IN, t=f}$	no unit	0.98	1
$\alpha_{OUT, t=f}$	no unit	0.98	1
$\alpha_{IN, t=0}$	no unit	0.98	1
$\alpha_{OUT, t=0}$	no unit	0.98	1

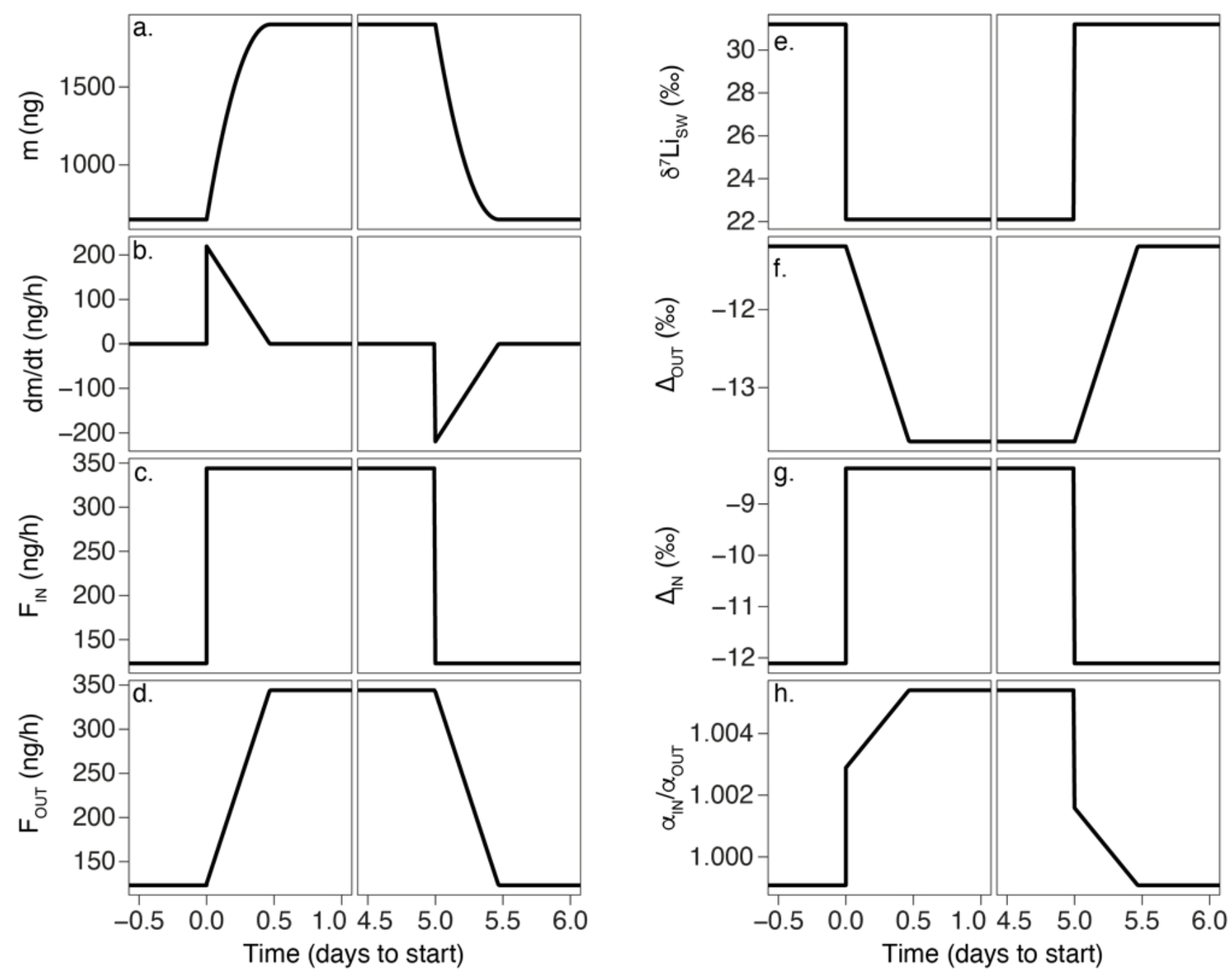

Figure S1: Example of modeled elemental and isotopic forcings from pre-experimental conditions to exposure (t = 0 to 5 days) and then depuration (t > 5 days): a. mass of Li in the mussel (ng), b. Li balance in the mussel (ng/h), c. Li influx (ng/h), d. Li efflux (ng/h), e. Seawater δ^7Li (‰), f. fractionation at excretion (‰), g. fractionation at intake (‰), h. ratio between intake and excretion fractionations (α_{IN}/α_{OUT})

Forward simulations

For every retained parameter set, we simulated coupled uptake and depuration and translated the resulting time-variable fluxes and isotope coefficients into isobxr-compatible scenario files. These scenarios formed the basis for the full suite of forward models of lithium cycling in mussels. The flux data generated by the model were then organized into worksheets using the ISOBXR_MASTER workbook (part of the isobxr package, Tacail 2021). A companion file, SCENARIO_MASTER, was used to define the sequence of model runs, the time steps, and the δ^7Li values for seawater. Each scenario was simulated using the sim.scenario function in the isobxr package.

Collating and filtering

For each lithium-cycle simulation scenario, we collated the model outputs, joined them with the corresponding parameter sets and run metadata, and retained only the simulations consistent with the experimental data within a two–standard error envelope.

Table S2: Descriptive statistics used for fitting simulations

Variable	Type	n	Average	SD	se
$[Li]_{MSL}$ (µg/g)	Control	48	0.90	0.22	0.05
$[Li]_{MSL}$ (µg/g)	Exposure	24	2.50	1.01	0.51
$[Li]_{MSL}$ (µg/g)	Depuration	24	0.93	0.26	0.13
$[Li]_{SW}$ (µg/g)	Control	4	0.18	0.00	0.00
$[Li]_{SW}$ (µg/g)	Exposure	24	1.65	0.00	0.00
$[Li]_{SW}$ (µg/g)	Depuration	24	0.18	0.00	0.00
BCF (no unit)	Control	48	4.75	0.89	0.18
BCF (no unit)	Exposure	24	1.52	0.62	0.31
BCF (no unit)	Depuration	24	5.16	1.44	0.72
δ^7Li_{MSL} (‰)	Control	12	31.95	0.67	0.27
δ^7Li_{MSL} (‰)	Exposure	24	30.24	1.90	0.95
δ^7Li_{MSL} (‰)	Depuration	24	32.10	2.38	1.19
δ^7Li_{SW} (‰)	Control	4	31.45	0.50	0.25
δ^7Li_{SW} (‰)	Exposure	24	22.06	0.00	0.00
δ^7Li_{SW} (‰)	Depuration	24	31.36	0.31	0.16
Δ^7Li_{SW-MSL} (‰)	Control	12	0.40	0.72	0.29
Δ^7Li_{SW-MSL} (‰)	Exposure	24	8.18	1.91	0.95
Δ^7Li_{SW-MSL} (‰)	Depuration	24	0.74	2.45	1.22
Mussel mass (g)	Control	48	0.90	0.05	0.03
Mussel mass (g)	Exposure	24	0.91	0.29	0.14
Mussel mass (g)	Depuration	24	0.84	0.35	0.18

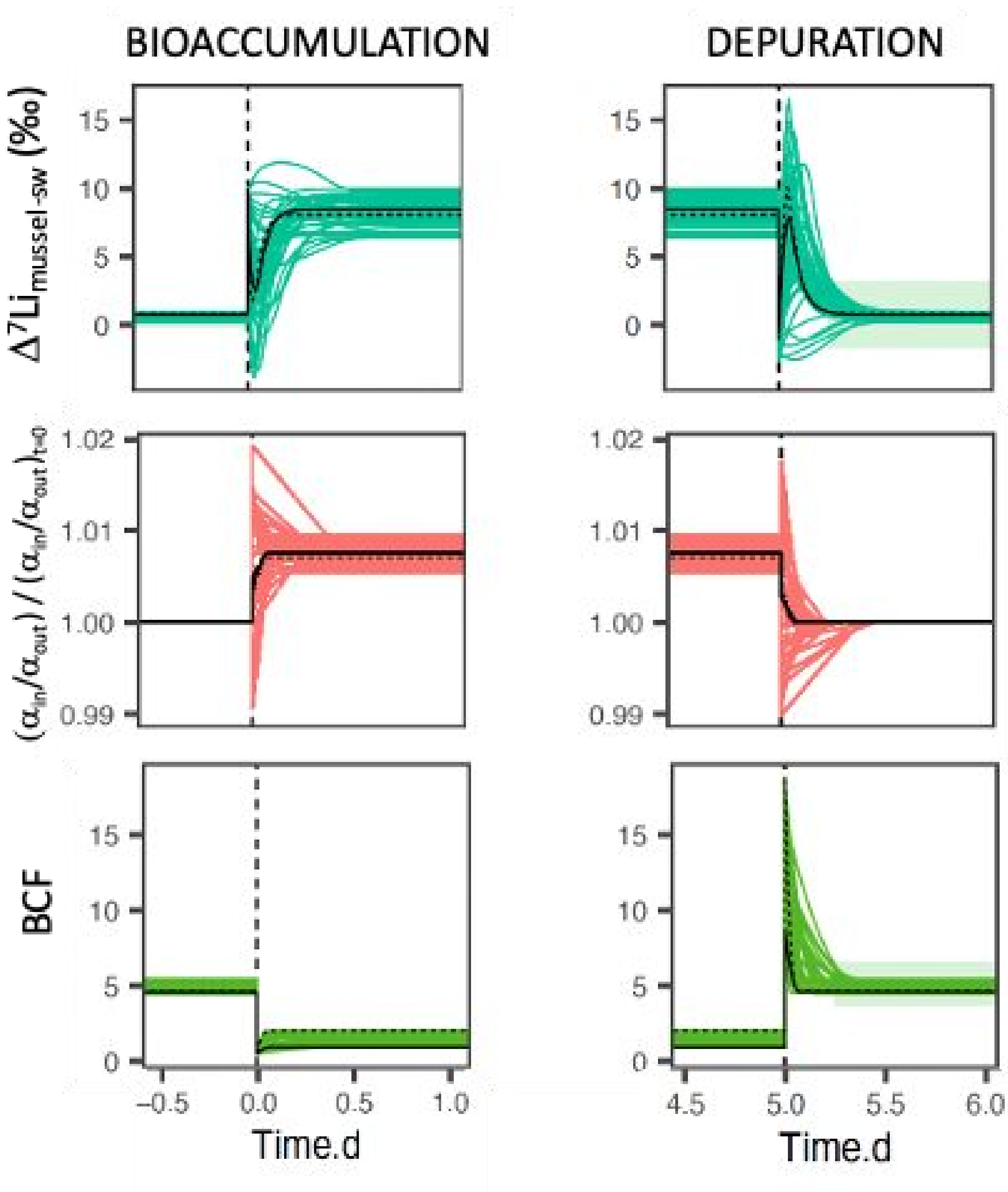

Figure S2: Modeling results for a selection of parameters. Other parameters are shown in figure 7 of the main text.
